# UK Reference Intervals for Parathyroid Hormone Using Abbott Methods

**DOI:** 10.3389/bjbs.2023.11224

**Published:** 2023-04-17

**Authors:** Mehdi Mirzazadeh, Craig Webster, Gayani Weerasinghe, Thomas Morris, Tim James, Brian Shine

**Affiliations:** ^1^ Department of Chemical Pathology, Epsom and St Helier University Hospitals NHS Trust, Carshalton, United Kingdom; ^2^ Department of Biochemistry, University Hospitals Birmingham NHS Foundation Trust, Birmingham, United Kingdom; ^3^ Department of Clinical Biochemistry, Buckinghamshire Healthcare NHS Trust, Amersham, United Kingdom; ^4^ Department of Clinical Biochemistry, Oxford University Hospitals NHS Foundation Trust, Oxford, United Kingdom

**Keywords:** age, gender, hyperparathyroidism, reference intervals, parathyroid hormone

## Abstract

**Background:** Diagnosis of hyperparathyroidism requires measurement of parathyroid hormone (PTH) in the context of the plasma calcium and other factors, such as vitamin D status and renal function. Accurate classification depends upon an appropriate population reference interval. We examined local population plasma PTH reference intervals at four different UK sites using a common platform.

**Methods:** Plasma PTH results were extracted from laboratory information systems at four different UK sites, all using the Abbott Architect i2000 method. We included only people with normal adjusted serum calcium, magnesium, vitamin D, and renal function. Following outlier rejection lower and upper reference limits were derived.

**Results:** An overall reference interval for plasma PTH of 3.0–13.7 pmol/L was observed using a non-parametric approach compared to 2.9–14.1 pmol/L using a parametric approach, notably higher than the manufacturer’s representative range of 1.6–7.2 pmol/L. We also noted statistically significant differences (*p* < 0.00001) between some sites with upper limits ranging from 11.5 to 15.8 pmol/L which may be due to different population characteristics of each group.

**Conclusion:** Locally derived reference intervals may be beneficial for UK populations and revised upper thresholds are necessary when using the Abbott PTH method to avoid inappropriate classification of patients as having hyperparathyroidism.

## Background

Parathyroid hormone (PTH) is secreted by the parathyroid glands. It controls plasma calcium concentrations by: increasing activation of vitamin D, leading to increased absorption of calcium from the gut; increasing reabsorption of filtered calcium in the renal tubular system; increasing release of calcium from bone by activation of the bone re-modelling system (1). Ionised calcium binds with the calcium sensing receptor (CASR) on the surface of parathyroid cells and inhibits secretion of PTH, so there is a reciprocal relationship between ionised calcium and PTH concentration in the blood (2). Disorders of excess PTH secretion (hyperparathyroidism) can be primary, for instance because of autonomous secretion by a tumour in one of the four glands, or secondary to a low calcium and/or low vitamin D. In primary hyperparathyroidism, the calcium is high and the PTH is not suppressed. In secondary hyperparathyroidism, the PTH is raised appropriately, to try to raise the calcium concentration into the reference interval. In hypoparathyroidism, damage to the parathyroid glands or a disorder of the CASR, leads to reduced plasma calcium (2).

Assessment of calcium disorders with a high or low calcium depends upon accurate measurement of PTH, and this, in turn, depends upon accurate knowledge of the “normal” PTH and thus of the reference interval (RI), the concentrations expected in healthy individuals without disorders of calcium metabolism. The RI can also be used to guide treatment in patients with chronic kidney disease.

The laboratories in our four hospital groups use the Abbott PTH assay, a method which is widely used across the United Kingdom (UK). The manufacturer recommends laboratories derive their own, local, RI but note a representative expected range in the instructions for use (IFU) of 1.6–7.2 pmol/L (3), values that are widely adopted in clinical practice. However, all our services have noted a high proportion of PTH results above the upper reference limit. This observation is consistent with a recent report from Italy in which a revised Abbott PTH RI based on 100 healthy blood donors of 2.7–11.6 pmol/L is presented (4). The same study also noted a RI derived from laboratory data of 2.0–11.9 pmol/L (4). We therefore examined data from four laboratory services within the UK to assess the consistency of PTH RI and how this compares to the manufacturers data.

## Methods

### Participating Sites

Four sites contributed data: Oxford University Hospitals NHS Foundation Trust (OUH), University Hospitals Birmingham NHS Foundation Trust (BIRM), Buckinghamshire Healthcare NHS Trust (BUCKS) and Epsom and St Helier University Hospitals NHS Trust (SHH).

### PTH Immunoassay

All sites used the i2000 Abbott Architect (Abbott Laboratories, Maidenhead, UK) intact PTH assay as recommended by the manufacturer. The method utilizes two goat polyclonal antibodies and has two running modes, one noted as “routine” and one “stat,” with the later mode allowing more rapid sampling and improved turnaround time. One site used the routine mode; two sites used the stat mode; and one switched between the two over the study period. Data from one site (SHH) showed no notable differences in results between the “routine” and “stat” operation. Imprecision was similar at each site, typical CV% being <8% below 10 pmol/L, <6% between 10 and 25 pmol/L and <5% at concentrations >25 pmol/L to 120 pmol/L consistent with the manufacturer’s reported performance characteristics in the IFU. All laboratories participated in the UK National External Quality Assurance Scheme (UKNEQAS) and had acceptable performance over the period that patient data was extracted. All sites used EDTA plasma specimens as recommended by the IFCC Scientific Division Working Group on PTH (5).

### Data Extraction

We used the laboratory information systems in the four laboratories to identify people who had had a PTH measurement. We included data from a 6-year period (2015–2021).

We included results from individuals aged 18 years and older, with estimated glomerular filtration rate (eGFR) more than 45 mL per minute per 1.73 m^2^ body surface area, adjusted calcium between 2.20 and 2.60 mmol/L, magnesium (6) more than 0.7 mmol/L (if recorded), and vitamin D concentration between 50 nmol/L and 200 nmol/L. Where people had multiple values, we used the first measurement.

The eGFR was calculated using the CKD-EPI (2009) (7) equation at all sites other than BHT who used the MDRD equation, taking into consideration plasma or serum creatinine concentration, age and sex, but not ethnicity, because this is not recorded in any of the laboratory information systems used by the four laboratories.

### Statistical Calculations

We used R (8) for all statistical analyses. We removed outliers using the Tukey method (9) on log-transformed values.

We derived the reference intervals from the mean ±1.96 standard deviations on the log-transformed values and back transformed the values (parametric approach) or by using the 2.5th and 97.5th centile values.

We examined values by month to see if there were annual trends.

## Results

**FIGURE 1:**
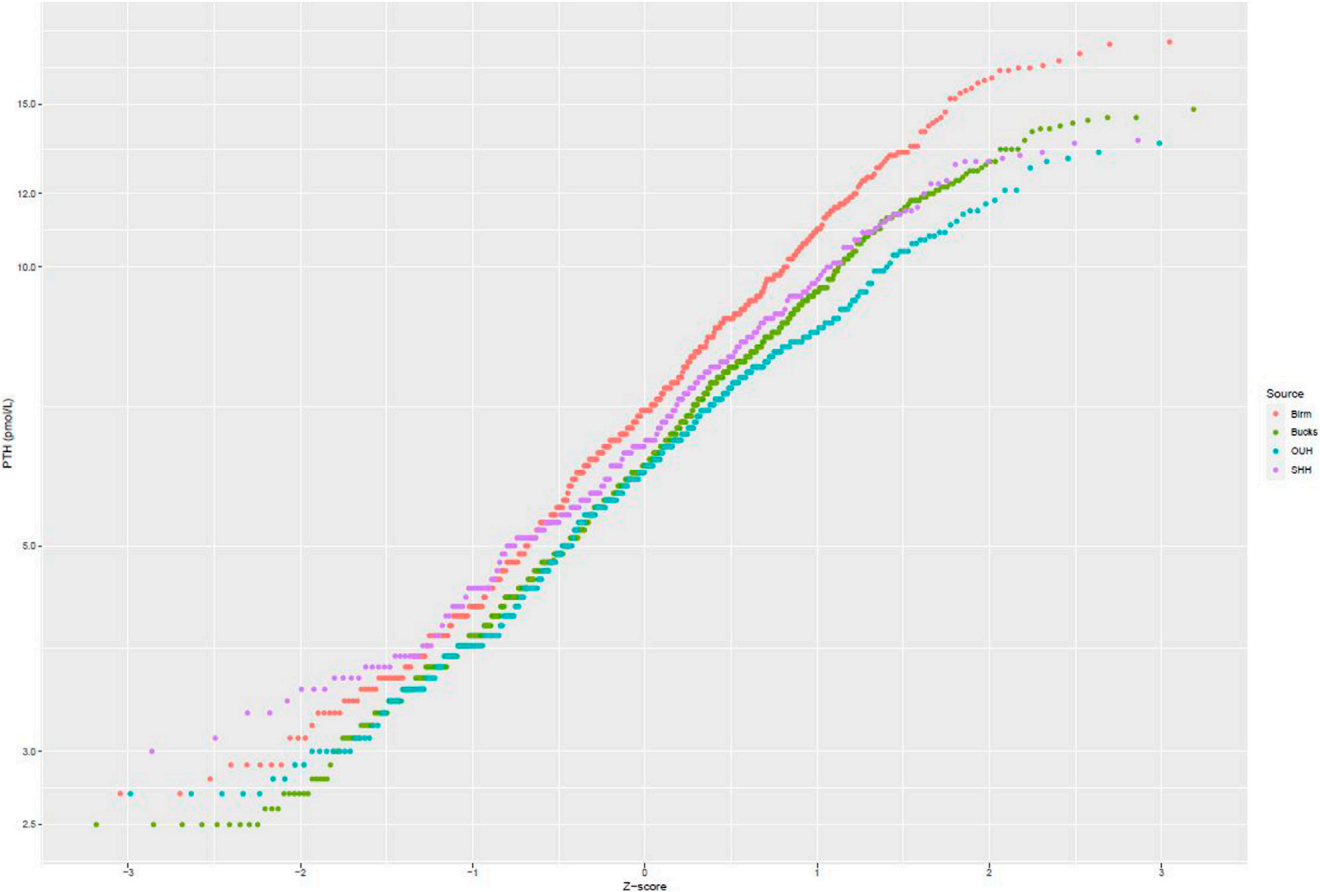
Probability plots of PTH concentrations (logarithmic scale) against theoretical z-scores by laboratory site.

After applying exclusion criteria to the downloaded data based on eGFR, adjusted calcium, magnesium and vitamin D, together with outlier identification, we used data from 1,727 people to calculate plasma PTH reference intervals. Probability plots (Figure 1) showed that these data had an approximately log-normal distribution. All the sites had different upper limits, some significantly different from each other (BIRM versus OUH and BUCKS, *p* < 0.0001; OUH versus SHH, *p* = 0.016, other differences not significant). The site-specific upper limits were all higher than those quoted by the manufacturer (Table 1) with an overall interval of 3.0–13.7 pmol/L (non-parametric derivation) and 2.9–14.1 pmol/L (parametric derivation).

**TABLE 1:** PTH Reference intervals by site.

Site	Number of tests	Gender	Median age	Non-parametric RI	Parametric RI
		%m/%f	Years (range)	pmol/L	pmol/L
Birmingham	432	23.8/76.2	63 (19–99)	3.2–15.8	3.0–16.1
Bucks	693	18.9/81.1	69 (20–98)	2.7–12.8	2.8–13.6
Oxford	356	34.3/65.7	50 (18–96)	3.0–11.5	2.9–12.3
St.Helier Hospital	238	21.8/78.2	69 (29–96)	3.5–13.0	3.2–13.3
**Overall (all sites)**	**1727**	**23.4/76.6**	**63 (18–99)**	**3.0–13.7**	**2.9–14.1**

**FIGURE 2:**
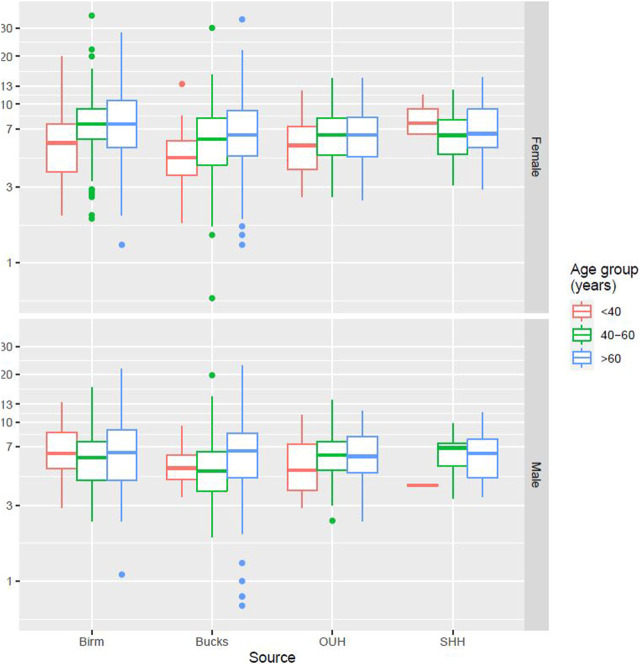
PTH distribution by age, gender, and laboratory site.

**FIGURE 3:**
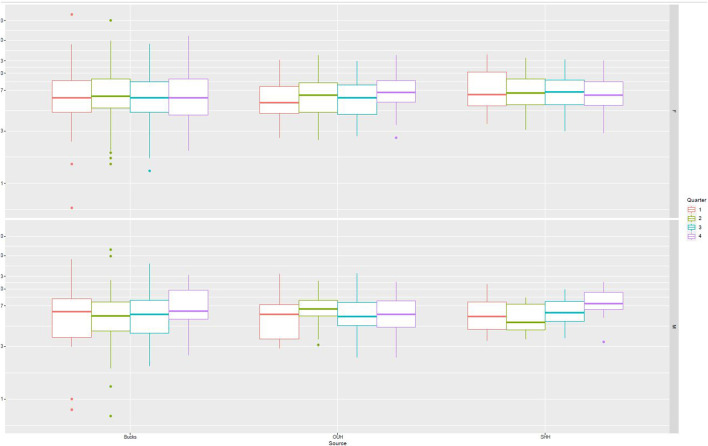
PTH distribution by quarter of the year.

Figure 2 presents data from all sites partitioned by age and gender. No specific patterns could be identified, and differences were not significant. Figure 3 presents data partitioned by quarter periods through the year. Again, no significant patterns were identified by time of year.

## Discussion

In this evaluation of service laboratory data, we have noted a difference between the representative values for plasma PTH, of 1.6–7.2 pmol/L, noted in the manufacturer’s IFU and those derived in local UK populations: all four sites had higher upper limits, using both parametric or non-parametric methods. Abbott state clearly in their IFU (3) for PTH that results at individual laboratories may vary and recommends determining a local range. Our observations that there are differences between sites and that these differ from those established in a group of healthy adults when undertaken by Abbott supports this suggestion. As a result of the data analysis and following consultation with service users, including endocrinology, each of our sites has adopted locally derived RIs to avoid misclassification of patients as having hyperparathyroidism and inappropriate follow up and investigation in adults.

Statistically different upper limits were noted between sites with a trend to higher upper RI in urban areas (Birmingham and South London/Surrey) compared to areas with more rural populations (Oxfordshire and Buckinghamshire). However, the differences between the upper limits for each site are small compared to the very large difference between the manufacturers upper limit and all sites assessed. The strict data exclusion criteria ensured only those with adequate vitamin D status, normocalcaemia, and adequate eGFR were included. The between site differences are likely to be due to the different population mix with respect to age and gender but may also be due to additional unidentified population related factors. It is possible that the ethnic mix of the different populations may be a factor in our observations. For example, it has previously been noted that one of the areas (BIRM) has a higher non-white population and higher indices of multiple deprivation (10).

A limitation of this study was the unavailability of patient ethnicity to allow this to be explored. The geographical difference noted requires further investigation but highlights the importance of locally determined ranges. UK government data from the Office for National Statistics (ONS) provides a breakdown of local population demographics and shows both variability between regions but that within any individual region this is relatively stable (11). This should mean the RIs derived should be applicable over the lifetime of the method and equipment in use.

The higher upper RIs found in this study are similar to those derived in an Italian study (4) where adult upper limits of 11.6 and 11.8 pmol/L were proposed.

Inaccurate reference intervals for any test have the potential for unnecessary follow up and intervention (12). PTH methods have developed considerably over the last 20 years with respect to specificity and the molecular form of the PTH molecule detected (13). It is therefore not surprising that laboratories are required to investigate and adopt adjusted RIs to reflect method performance. Adoption of the updated ranges presented in the present study should improve appropriate investigation of abnormal PTH values.

We excluded data from those less than 18 years of age to enable an adult range to be derived. The data available on children was limited and therefore we were unable to explore the reference range in paediatrics as the data sets were considered too small for valid partitioning across childhood. The CALIPER study (14) has established PTH intervals for children on instruments from several major manufacturers, including the Abbott i2000 instrument used in our laboratories. The reference intervals quoted are lower than those we have determined in the present study for adults: ages 6 days to <1 year being 0.68–9.39 pmol/L; from 1 to <9 years: 1.72–6.68 pmol/L; 9 to <17 years: 2.32–9.28 pmol/L; 17 to <19 years: 1.70–6.40 pmol/L.

A potential limitation in the selection of results for data analysis was that not all patients had a serum magnesium result available to allow exclusion of those with abnormal levels. This could have therefore biased the data set through inclusion of PTH results from patients who had low or high magnesium results. However, we do not consider this would have had a major impact on the data analysis. A further consideration is reagent and calibrator usage including lot number changes and how these might contribute to result bias. The laboratories involved have reagent acceptance procedures in place to fulfil accreditation requirements, and acceptable IQC and EQA over the study period. These quality management procedures mitigate the potential impact of lot number changes on the derived RIs, as does the use of data collected across more than a full calendar year within which there would be several lot number changes.

In conclusion, there appears to be good evidence that a locally derived plasma PTH RI is required for an adult UK population to prevent inappropriate patient anxiety and follow up.

## Summary Table

### What is Known About This Subject

• Parathyroid hormone (PTH) is analysed in clinical laboratories to identify hyperparathyroidism, a common endocrine abnormality.
• Identification of patients with hyperparathyroidism is only possible if accurate PTH reference intervals are applied.
• PTH reference intervals can vary between the different methods used in practice.

### What This Paper Adds

• When measured with the Abbott method PTH values in UK populations have upper limits higher than those commonly used in practice.
• PTH distributions in four UK sites using the Abbott method all had higher upper limits than the commonly used range.
• Application of revised reference intervals for PTH measured with the Abbott method will avoid unnecessary follow up of patients.

## Concluding Statement

This work represents an advance in biomedical science because as it provides a revised UK reference interval for PTH when measured using the Abbott method and therefore improves its diagnostic accuracy for hyperparathyroidism.

## Data Availability

The data that support the findings of this study are available from the corresponding author, but restrictions apply to the availability and an agreed process for access would be required.
